# Recent Advances in Therapeutic Drug Monitoring of Voriconazole, Mycophenolic Acid, and Vancomycin: A Literature Review of Pediatric Studies

**DOI:** 10.3390/pharmaceutics13121991

**Published:** 2021-11-23

**Authors:** Matylda Resztak, Joanna Sobiak, Andrzej Czyrski

**Affiliations:** Department of Physical Pharmacy and Pharmacokinetics, Poznan University of Medical Sciences, 6 Święcickiego Street, 60-781 Poznań, Poland; jsobiak@ump.edu.pl (J.S.); aczyrski@ump.edu.pl (A.C.)

**Keywords:** therapeutic drug monitoring, voriconazole, mycophenolic acid, vancomycin, children

## Abstract

The review includes studies dated 2011–2021 presenting the newest information on voriconazole (VCZ), mycophenolic acid (MPA), and vancomycin (VAN) therapeutic drug monitoring (TDM) in children. The need of TDM in pediatric patients has been emphasized by providing the information on the differences in the drugs pharmacokinetics. TDM of VCZ should be mandatory for all pediatric patients with invasive fungal infections (IFIs). Wide inter- and intrapatient variability in VCZ pharmacokinetics cause achieving and maintaining therapeutic concentration during therapy challenging in this population. Demonstrated studies showed, in most cases, VCZ plasma concentrations to be subtherapeutic, despite the updated dosages recommendations. Only repeated TDM can predict drug exposure and individualizing dosing in antifungal therapy in children. In children treated with mycophenolate mofetil (MMF), similarly as in adult patients, the role of TDM for MMF active form, MPA, has not been well established and is undergoing continued debate. Studies on the MPA TDM have been carried out in children after renal transplantation, other organ transplantation such as heart, liver, or intestine, in children after hematopoietic stem cell transplantation or cord blood transplantation, and in children with lupus, nephrotic syndrome, Henoch-Schönlein purpura, and other autoimmune diseases. MPA TDM is based on the area under the concentration–time curve; however, the proposed values differ according to the treatment indication, and other approaches such as pharmacodynamic and pharmacogenetic biomarkers have been proposed. VAN is a bactericidal agent that requires TDM to prevent an acute kidney disease. The particular group of patients is the pediatric one. For this group, the general recommendations of the dosing may not be valid due to the change of the elimination rate and volume of distribution between the subjects. The other factor is the variability among patients that concerns the free fraction of the drug. It may be caused by both the patients’ population and sample preconditioning. Although VCZ, MMF, and VAN have been applied in pediatric patients for many years, there are still few issues to be solve regarding TDM of these drugs to ensure safe and effective treatment. Except for pharmacokinetic approach, pharmacodynamics and pharmacogenetics have been more often proposed for TDM.

## 1. Introduction

Therapeutic drug monitoring (TDM) is generally defined as a measurement of the drug concentrations in order to optimize dosage and individualize therapy. It should be emphasized that drug concentration’s measurement should not be used as the only method of therapy optimization. The priority is to achieve a therapeutic effect without side effects of the drug. However, it is challenging to find the adequate dosages regimens to meet these conditions.

TDM is used mainly for the management of drugs with a narrow therapeutic index, large pharmacokinetics variability, and a reasonable correlation between the drug concentration and the clinical effects. Both voriconazole (VCZ) and mycophenolic acid (MPA) as well as vancomycin (VAN) meet these criteria.

As there is usually no information on the individual pharmacokinetic parameters of the patient at the start of treatment, reference is made to published recommendations. All subsequent dose adjustments should be made based on the concentration measured and the patient’s clinical response.

Application of TDM in routine clinical practice is essential in a specific group of patients such as children. This population demonstrates wide intersubject variability of pharmacokinetic parameters associated with physiological changes during growth and maturation. Additionally, polytherapy, genetic polymorphism, and various serious underlying conditions impact unpredictable dose–exposure relationships to result in treatment failure. For this reason, the number of studies on the pharmacokinetics/pharmacodynamic (PK/PD) of drugs in children has increased significantly in recent years.

In the present review, we focus on TDM of the chosen representatives of antifungal, immunosuppressants, and bactericidal drugs. This article presents recent advances regarding TDM of the abovementioned drugs in pediatric patients. The aim of this review is to more precisely define the impact of pharmacokinetics on TDM and efficacy and toxicity of VCZ, MPA, and VAN treatment.

## 2. Voriconazole (VCZ)

### 2.1. Useful PK/PD Parameters

VCZ is a second-generation triazole agent with potent spectrum antifungal activity against *Aspergillus* species, *Candida* species, and molds that other triazoles are resistant to [1]. In clinical settings, VCZ is the drug of the first choice for the treatment of invasive fungal infections (IFIs) in immunocompromised children with hematologic malignancies and allogeneic hematopoietic stem cell transplantation (allo-HSCT) [2]. However, incomplete response to therapy and toxicity can cause ineffective treatment of IFIs [3]. VCZ is available for clinical use in both adults and children aged 2 years and older as a lyophilized powder for intravenous infusion (IV) and as coated tablets, as well as powder for suspension for oral administration (PO) [4,5]. VCZ drug plasma concentrations are unpredictable. Several factors including nonlinear pharmacokinetics, age, body mass index (BMI), cytochrome P450 2C19 polymorphism, drug–drug interaction, and variable oral bioavailability particularly in children are responsible for large inter- and intrapatient variability of VCZ pharmacokinetics (PK) [6].

As a result, it is challenging to optimize daily dosing to achieving therapeutic range in pediatric patients. TDM is recommended throughout treatment to optimize efficacy and minimize adverse reactions [7,8].

The efficacy of VCZ depends on the minimal inhibitory concentration (MIC) of the drug against the pathogen. A preclinical model of disseminated *Candida albicans* infection has shown that the ratio of the area under the curve (AUC) to MIC (AUC/MIC) is the best predictor of VCZ efficacy [9]. Wang et al. concluded that the free AUC/MIC ratio was higher in 25 predicted clinical responses in IFIs patients [10,11]. Unfortunately, these measurements are difficult to perform in clinical practice, especially in children. Consequently, trough concentrations (C_trough_) are a practical alternative in PK studies of VCZ [11].

### 2.2. Timing of Initial TDM and Target Concentration of VCZ

The initial TDM should be performed as early as possible. The C_trough_ should be monitored at steady state after 5–7 days of treatment with maintenance dosing. However, when a loading dose is administered, steady state is achieved earlier, and a C_trough_ blood sample can be taken from 3–5 days of therapy [12,13].

A C_trough_/MIC ratio of 2 and 5 or a C_trough_ >1 mg/L was reported as an efficacy target and a C_trough_ <4–6 mg/L as a safety target for VCZ [14,15,16]. However, the optimal therapeutic VCZ range is still not clearly defined [17]. Numerous observational studies have recommended the therapeutic range of VCZ as 0.5–4.0 mg/L [18], 1.0–5.0 mg/L [19], 1.0–5.5 mg/L [20,21], 1.0–6 mg/L [22], and 1.5–4.0 mg/L [23]. However, most studies recommended a C_trough_ level between 1 and 5.5 mg/L.

Neely et al., who evaluated 46 patients (0.8–20.5 years) in a single-center retrospective study, showed that each VCZ serum C_trough_ of <1 mg/L was significantly associated with a 2.6-fold increased risk of death (*p* = 0.002) [24]. Similarly, a relationship between outcome and subtherapeutic C_trough_ of VCZ was reported in other TDM studies [25,26]. Choi et al. demonstrated that treatment failure was more frequently observed in children with C_trough_ <1 mg/L (success vs. failure, 19.7% vs. 42.1%) [25]. Kang et al. found at 12 weeks of therapy that the poor outcome in the non-TDM group was higher than the TDM group (78.6% vs. 40.0%) [26]. Recently, Hanai et al. designed a systemic review and meta-analysis to determine the optimal trough level of VCZ in the pediatric population. In this study, 211 children with hematologic disorders, solid organ transplantation, and from Asian and non-Asian countries were included. The authors found that the efficacy of IFIs treatment increases significantly with cutoff values of ≥1.0 mg/L [27].

High plasma concentration of VCZ might be associated with an increased risk of toxicity. As VCZ is well tolerated in children with a lower rate of adverse effects (AEs), toxicity targets have not been validated for this population so far [27]. However, several studies have reported the incidence of adverse drug reactions. The most common AEs included hepatotoxicity [8,20,28,29,30,31,32], phototoxicity [28,29], and visual disturbance [30].

A significant relationship between VCZ plasma concentration out of range >5.5 mg/L and neurological and skin toxicity (*p* = 0.0001) was established by Soler-Palacín [11]. It is important to note that AEs occur even when VCZ plasma levels are within the therapeutic range as shown by Boast et al. [29]. In their TDM study, performed on 55 children with IFIs, hepatotoxicity occurred in 12.7% of patients, and none of them had VCZ serum levels >5 mg/L. Similar observations were presented in the study conducted by Gerin et al. [31]. Hepatotoxicity was reported in 4 children and VCZ was discontinued in 3 of them. All observed VCZ C_trough_ values were <5.5 mg/L.

There are major differences between the PK of VCZ in children and adults. The interindividual PK variability appears to be larger in the pediatric population due to faster weight-normalized clearance rate, greater systemic and first-pass metabolism compared with adults [24,25,32]. The oral bioavailability of VCZ in children is equal to 62% and is lower than in adults [28,33]. Additionally, dose-dependent VCZ PK were observed in a pediatric population. Linear PK was reported with VCZ doses between 3 to 4 mg/kg every 12 h while nonlinear PK occurred at VCZ doses from 7 to 8 mg/kg every 12 h [6,17].

### 2.3. The Optimal Dosage Regimen

Dose recommendation in children changed in 2012, based on PK studies reporting inadequate drug exposure. However, despite dose increasing, many investigators still observe a high proportion of patients with VCZ below the therapeutic range. Barteling et al. evaluated 92 HSTC pediatric patients between 0 and 20 years of age treated with VCZ [3]. It was found that regardless of age and administration route, only 34% of these patients reached C_trough_ VCZ level within the proposed therapeutic range of 1 to 5 mg/L. In addition, 56% of those patients had C_trough_ of VCZ <0.5 mg/L. TDM study performed by Tucker et al. in 11 pediatric oncologic/bone marrow transplant patients with a median age of 8 confirmed earlier reports [34]. Authors found that only 50% of patients achieved C_trough_ of VCZ with the recommended dosage median of 6 mg/kg every 12 h. The rest of the patients required higher doses or more frequent dosage intervals to achieve target VCZ concentration. Mori et al. provided information on VCZ PK in Japanese children [35]. The number of 24 patients from 0 to 17 years old with a median VCZ doses of 7.6 mg/kg and 8.1 mg/kg twice daily for IV and PO administration, respectively, were investigated. In this study, large variability in C_trough_ was also observed. The range VCZ C_trough_ following PO administration was 0.11–6.19 mg/L and was comparable with that following IV infusion which was 0.13–5.19 mg/L. Half of the measured C_trough_ values did not reach the 1 mg/L target. Another group of researchers used TDM to guide the VCZ dosing strategy in patients <2 years [36]. Although the VCZ does not have an official recommendation for children <2 years of age, it is used off-label in newborns in special cases [37]. Initial dose of VCZ for 3 patients was 9 mg/kg intravenously/enterally twice a day. Sets of VCZ C_trough_ were obtained and all were subtherapeutic (0.31–0.89 mg/L). The authors suggested higher initial doses with 3 times/day dosing schedule to achieve VCZ therapeutic level. Doby et al. presented data from 10 pediatric patients aged <3 years treated with VCZ of dose 9 mg/kg/dose every 12 h who underwent TDM [38]. It was found that C_trough_ ranged from 0.1–3.2 mg/L and only 3 (18%) C_trough_ values >1 mg/L. The authors concluded that higher doses are needed in young children to achieve target concentrations. The study performed by Boast et al. for 55 patients showed that children <6, 6 to 12, and >12 years of age needed median IV doses of 8.8, 7.5, and 4.0 mg/kg every 12 h, respectively (*p* < 0.001) [29].

Koto et al. performed a TDM study of VCZ in 20 children aged <8 years after stem cell transplantation [8]. The recommended maintenance dose regimen with a median of 10.5 mg/kg/day was used. It was concluded that more than half of the children did not achieve C_trough_ of 1.0 mg/L at the first and second monitoring of the drug. The authors also showed that children aged <5 years had significantly lower C_trough_ of VCZ in comparison with patients aged 6–12 years. It was reported that higher doses are required in younger patients particularly those receiving PO administration. Another study in the Asian pediatric population was conducted by Kang et al. [26]. The number of 61 children <19 years old with hemato-oncological diseases and organ transplantations were divided into TDM (*n* = 31) and non-TDM (*n* = 30) group. The authors found that only 49.4% of C_trough_ levels were within the therapeutic range. This study also confirmed that children <12 years old require higher doses to achieve VCZ target compared with those >12 years of age (8.3 mg/kg and 6.9 mg/kg every 12 h, respectively).

Most of the TDM VCZ studies were carried out on small groups of children and it was challenging to evaluate statistically significant correlations. Allegra et al. reported a TDM study from 237 children [39]. In all 237 enrolled patients, high interindividual variability of C_trough_ was proved (range 0.69–3.18 mg/L). For children who received IV administration, and with higher serum creatinine levels, higher C_trough_ were found (*p* < 0.001). A positive and significant correlation between C_trough_ and age was observed (*p* = 0.036). In patients receiving PO administration, the authors observed a positive correlation between VCZ dose and drug plasma exposure (*p* < 0.001). Moreover, they concluded that sex significantly influenced VCZ levels, with a higher median drug concentration for the male group (*p* < 0.001). A statistically significant group of children competed in a study by Liu et al. [2]. The authors evaluated 107 pediatric patients aged 0.1–11.1 years. In this study, the dose–exposure correlation was investigated and appropriate initial dosing regimens were evaluated. Similar to published results, high VCZ C_trough_ variability especially in patients aged <2 years and those aged 2–12 years was observed. They found that only 47.7% of patients reached the VCZ therapeutic level of 1.0–5.5 mg/L. In their opinion, in a group aged <2 years dose of 5 to <7 mg/kg significantly increased the chance to reach the target of VCZ concentration compared with the 3 to <5 mg/L dose recommended.

Recent studies also showed that the recommended dosages did not lead to adequate therapeutic drug levels in the majority of children. Lampers et al., in a study with a cohort of 21 pediatric patients, reported a high percentage of VCZ C_trough_ <1 mg/L [5]. Upon first measurement, only 52.4% of children reached C_trough_ between 1–6 mg/L. The dose adaptations were needed to achieve VCZ therapeutic levels. Hu et al. reported that Asian children required lower doses of VCZ than the recommended to achieve the target range of 1.0–5.5 mg/L [20]. Authors observed that only 50% of children (21/42) aged 2–14 years old had initial C_trough_ ≥1 mg/L with a range from 0.02–9.35 mg/L. However, only 28% of those patients had the dose adjustment. The average oral and IV adjusted doses were 7.7 mg/kg and 5.6 mg/kg twice a day to achieved VCZ therapeutic levels. Zhao et al., in a TDM study of children aged 1 to <18, concluded that patients <12 years tended to have a higher intraindividual PK variability [40]. More than 50% of 94 pediatric patients did not reach the VCZ therapeutic range at the initial C_trough_ (ranged from 0.04 to 16.11 mg/L). The younger children, ≤12 years, obtained significantly lower C_trough_ (*p* = 0.0096). The median dosage needed to achieve adequate prophylaxis and treatment of VCZ by age group was additionally investigated by Duehlmeyer et al. [41]. It was found that only 50% of VCZ C_trough_ levels for 107 children were considered within the therapeutic range. The authors established the median dose by age ≤6, 7–12.9, ≥13 as follows: 8.1, 6.7, and 3.3 mg/kg. Recently, another TDM study including 27 children aged 2–12 years, was performed [42]. The authors aimed to evaluate VCZ monitoring in children with IFIs after the administration of new dosages. It was found that new dosing led to a higher rate of adequate levels of VCZ, compared to previous studies. However, still, 35.8% of VCZ C_trough_ were outside the therapeutic range; 27.5% were subtherapeutic and 8.3% were supratherapeutic. The authors concluded that the estimated dose was significantly higher in children <8 years than in those ≥8 years (21 vs. 16.5 mg/kg/day; *p* = 0.02).

### 2.4. Factors Affecting Serum Concentration in TDM

A growing number of studies have documented that CYP2C19 polymorphism makes a crucial contribution to the extensive pharmacokinetic variability of VCZ [4]. VCZ is metabolized by primary by liver through CYP2C19 and to a lesser extent CYP2C9 to its main circulating N-oxide metabolite. CYP2C19 phenotype variants are known to be strongly correlated with differences in the therapeutic plasma concentration of VCZ [43]. Genetic polymorphism of CYP2C19 is associated with 30% to 50% variation in VCZ metabolism between individuals [44]. The wild-type allele CYP2C19*1 encodes normal function and homozygous (CYP2C19*1*1) considered as the normal metabolizers (NMs). The notable functional allelic variants known to impact VCZ pharmacokinetics are CYP2C19*2, *3, and *17. CYP2C19*2, and CYP2C19*3 homozygous alleles are classified as poor metabolizers (PMs) phenotype while heterozygous alleles are classified as intermediate metabolizers (IMs) phenotype. Patients with CYP2C19*17 allele are classified as ultrarapid metabolizers (URMs) phenotype [4,45,46]. The proportions of CYP2C19 PMs vary with race ethnicity. It has been shown that PMs phenotype is present in only about 3–7% of Caucasians and African Americans and 12–23% of Asians population, with a higher frequency in Japanese than in Chinese patients (21.3% vs. 13.7%, respectively) [35,45,47].

Limited data on the impact of CYP2C19 genetic variants on the VCZ pharmacokinetics in pediatric patients are available. Hick et al. elaborated the impact of CYP2C19 genotypes on VCZ plasma concentrations in 33 immunocompromised children (aged 1–19 years) [48]. VCZ C_trough_ between 1 and 6 mg/L were considered to be therapeutic. The authors found that CYP2C19 genotypes were associated with VCZ C_trough_ (*p* = 0.002). VCZ dose normalized plasma C_trough_ were significantly lower in URMs phenotype patients (median 0.01 mg/L/mg/kg; *p* = 0.04) and significantly higher in PMs phenotype patients (median 0.62 mg/L/mg/kg; *p* = 0.04); than in EMs phenotype patients (median 0.07 mg/L/mg/kg). It was shown that children who had URMs phenotype never achieved VCZ target range at any dose. The authors concluded that CYP2C19*17 homozygous pediatric patients would require higher doses to reach the VCZ target concentrations. Another analysis, performed by Narita et al., in 37 Japanese patients in the pediatric group (aged 1–15 years) showed that C_trough_ of VCZ were significantly higher (*p* = 0.004) in the PMs and IMs metabolizer groups compared with the EMs metabolizers and URMs groups; medians were 0.54 and 0.09 mg/L, respectively [43]. The authors concluded that in patients with VCZ C_trough_ lower than the target range, daily doses had to be increased according to reports from western countries. Teusing et al. analyzed 20 children with a median age of 10.9 years undergoing HSCT who received CYP2C19 genotype-directed dosing [49]. Results showed a significant difference in doses and time required to achieve C_trough_ of VCZ for different CYP2C19 genotypes. The algorithm according to the individual CYP2C19 genotype was performed. The authors found that in the follow-up study, no patients who had C_trough_ lower than <1 mg/L and higher than >5.5 mg/L and showed a statistically significant (*p* < 0.001) decrease in time to reach the VCZ target range of 1–5.5 mg/L from 29 to 6.5 days when genotype-guided dosing was used.

Due to the fact that VCZ is an inhibitor and a substrate for CYP2C19, 2C9, and 3A4, drug–drug interactions with agents metabolized by these pathways are common. Coadministration of these drugs could be a rationale for VCZ TDM. Liu et al. reported that omeprazole significantly increased the C_trough_ of VCZ in pediatric patients (*p* = 0.032) [2]. Similar findings were also observed in a study by Hu et al. [20]. Proton pump inhibitors (PPIs) significantly increased C_trough_ VCZ compared to those with VCZ administration only (*p* = 0.028). On the other hand, Spriet et al. did not find a significant impact of omeprazole on VCZ PK (*p* = 0.78) [28]. The following drug–drug interaction was reported by Soler-Palacín [11]. Low plasma VCZ levels were observed until carbamazepine was discontinued from treatment. In recent studies on the pediatric population, the association between C-reactive protein levels (CRP) and C_trough_ was observed [42,50,51]. Luo et al. reported a significant correlation of CRP and C_trough_ VCZ for children in age 11–18 years but not in the age of 2–10 years.

Serum albumin levels decrease in critically ill patients with chronic infection. Hypoalbuminemia is known, usually resulting in higher free drug concentration in plasma, and in that case, the effect of the drug may be increased. The impact of hypoalbuminemia on C_trough_ VCZ in pediatric patients was found [2,42]. The authors suggested that optimizing albumin levels could maximize the treatment effect. In another study, Vanstraelen et al. concluded that the measured VCZ total concentration should be interpreted with caution, especially in patients with hypoalbuminemia and VCZ C_trough_ close to the upper limit of the therapeutic range [52]. The authors postulated to consider dose adjustments for patients with adverse events potentially related to an increased unbound plasma concentration. Additionally, the simultaneous measurement of both the total and unbound VCZ concentration might provide a more complete understanding of VCZ PK variability observed in patients [52,53].

### 2.5. The Dosing in Different Groups of Patients

Age is one of the most important factors influencing VCZ plasma exposure, as VCZ clearance was shown to be much higher in children under the age of 12, and oral bioavailability of VCZ is lower in children compared to that in adults [5]. Early published data suggest that the recommended dosages for pediatric patients were inadequate to achieve C_trough_ greater than 1 mg/L, especially for younger children (<12 years) [28,32,34,54]. In the study performed by Spriet et al., 10 children (0.9–18 years of age) with IV and PO median dose of 6.95 mg/kg every 12 h, were examined. The authors confirmed that VCZ levels varied widely in children; they ranged from 0.09 to 4.90 mg/L <12 years of age and from 0.11 to 1.71 mg/L in patients >12 years old. They found that levels of VCZ C_trough_ were within the therapeutic range in only one-third of cases. No correlation between C_trough_ and administered dosage was observed. They concluded that starting with IV dose of VCZ at least 7 mg/kg every 12 h and performing TDM regularly may guarantee the effectiveness of therapy. Higher daily dosages were suggested by Gerin et al. [31]. The authors evaluated C_trough_ of VCZ obtained from 6 infants and 10 children after IV administration. In these groups, C_trough_ <1 mg/L was observed in 77% and 47% cases, respectively. The authors found that increased daily dosages in the range of 20–32 mg/kg in some pediatric patients were necessary to achieve C_trough_ >1 mg/L. This was consistent with the findings of Brüggemann et al., who performed TDM practice in 18 children aged from 0 to 18 years [55]. In 44% of patients, the first C_trough_ of VCZ was lower than the therapeutic target. Choi et al., in TDM studies of 27 patients <19 years, demonstrated a significant correlation between PO doses and C_trough_ of VCZ in patients ≤6 years old (*p* = 0.027) [25]. It was found that younger children needed higher PO doses compared to older pediatric groups (median dose of 8.9 vs. 4.2 mg/kg every 12 h, *p* < 0.001). In another study, Soler-Palacín et al., evaluated 30 immunocompromised children aged 0–17, who were treated with VCZ at a median dose of 20 mg/kg/day [11]. As much as 50% of the C_trough_ VCZ plasma samples were reported as <1 mg/L. Finally, a total of 73% of patients required a dose adjustment. The authors concluded that patients <5 years of age needed a median dose of 38 mg/kg/day while patients >5 years of age median dose of 15 mg/kg/day to achieve VCZ therapeutic levels. Similarly, the study conducted by Pieper et al. showed that the recommended doses were too low to achieve adequate VCZ levels in younger children [56]. In a group of 72 children (0.2–18 years of age) with a median maintenance dosage of 4.8 mg/kg twice daily, C_trough_ of VCZ were within a range of <0.2 to 14.9 mg/L, and no correlation to dose was observed (*p* = 0.07). Of the samples, in 22%, 42%, and 58%, VCZ concentrations were <0.2, ≤0.5, and ≤1.0 mg/L, respectively. Dosage modification was needed in 31 cases.

High variability of the VCZ PK is also observed in patients with chronic liver dysfunction [57]. A study performed by Wang et al. in adult populations demonstrated that clearance (CL) of VCZ decreased with increasing severity of hepatic failure according to the Child–Pugh classification [58]. Patients with mild to moderate (Child–Pugh Class A and B) liver cirrhosis demonstrated higher plasma exposure of VCZ compared with those with normal hepatic function. Thus, the maintenance dose should be halved in those patients. Additionally, Lin et al. noted an increase of VCZ terminal half-life of about five times in Child–Pugh Class C adult patients [59]. Due to no dosage recommendation of Class C patients, the authors suggested closely monitoring VCZ to adjust appropriate dosage based on the obtained results to avoid serious adverse events. To the best of our knowledge, such studies have not been conducted in the pediatric population.

### 2.6. Additional Information Useful for VCZ TDM

The success of VCZ TDM depends on the quality of analytical methods used for the determination of VCZ concentrations in biological fluids. Numerous HPLC methods with UV, FLD, and MS detection have been presented as common in VCZ TDM [7,53,60,61]. The extremely important aspect of the TDM practice, especially in pediatric patients, is the amount of sample that is required for analysis. HPLC-MS methods are attractive because they accept a small sample size, even 50 μL [62]. Another point to consider is the sample pretreatment procedure. It is important to simplify the analytical procedure so that it can be successfully used in TDM. Some published HPLC methods still apply liquid–liquid extraction [57]. However, those methods are time-consuming and may cause errors resulting from changes in extraction efficiency. Some authors suggested an alternative technique of VCZ extraction based on one-step plasma protein precipitation, which are faster and easier to perform [53,63].

## 3. Mycophenolic Acid (MPA)

### 3.1. MPA Characteristics

In children treated with MPA, similarly as in adult patients, the role of TDM for MPA has not been well established and is still undergoing continued debate, whereas TDM for other immunosuppressants such as cyclosporine (CsA), tacrolimus (Tac), or sirolimus has already been implemented [64,65]. MPA was firstly registered in prevention of acute rejection after renal transplantation; however, its activity is used not only after solid organ, intestinal, and HSCT but in other diseases, mainly of autoimmune background (lupus nephritis, nephrotic syndrome) as well. MPA TDM is recommended in pediatric patients, but it is still not a routine practice although numerous studies proved the advantage of dose adjustment based on TDM [66,67,68,69,70,71,72,73]. Our review includes studies on the MPA TDM in children after renal transplantation [67,74,75,76,77,78,79,80,81], other organ transplantation such as heart [82,83], liver [72], or intestine [65], children after hematopoietic stem cell transplantation [84,85,86,87,88,89] or cord blood transplantation [90], and children with lupus [71,73,91,92,93,94,95,96], nephrotic syndrome [69,70,97,98,99,100,101,102,103,104,105,106,107], Henoch-Schönlein purpura [108], and other autoimmune diseases [109].

MPA is an active form of mycophenolate mofetil (MMF). It is also administered as enteric-coated mycophenolate sodium (EC-MPS); however, the usage of this formulation is limited in pediatric patients [66]. MPA is a selective, noncompetitive, and reversible inosine monophosphate dehydrogenase (IMPDH) inhibitor [65]. IMPDH is the rate-limiting enzyme in de novo synthesis of guanosine nucleotides. In this manner, MPA suppresses cell-mediated immune responses and antibody formation by inhibiting the proliferation of T and B-lymphocytes, which are more dependent on the novo pathway than other cell types [110,111]. MPA is characterized by considerably mild adverse effects if compared with other immunosuppressants (e.g., CsA, Tac). The main obstacle during MMF treatment is the variability of its pharmacokinetics [67], which precludes obtaining the efficient MPA concentrations in plasma.

### 3.2. Useful PK/PD Parameters

MPA C_trough_ is not a good surrogate for its overall exposure [87,98] and does not correlate with clinical outcomes in pediatric renal transplant recipients [75]. One of the factors that enables using C_trough_ in MPA TDM is probably concomitant CsA administration as CsA inhibits MPA enterohepatic recirculation [65,110]. In children treated with MMF and Tac, the contradictory observation was made by Todorova et al. [112], who found a statistically significant correlation between MPA exposure and MPA trough levels supporting the use of trough levels to monitor exposure. Generally, MPA TDM should be based on MPA exposure, which is expressed by the area under the concentration-time from 0 to 12 h curve (AUC_0–12_). In children treated with MMF due to different than renal transplantation indication, MPA C_trough_ is frequently also analyzed in relation to TDM.

Whereas TDM based on C_trough_ would be easier and more convenient, MPA AUC_0–12_ estimation is difficult to perform in clinical practice, as it requires multiple blood sampling within 12 h [71]. The approach that facilitates the MPA exposure estimation by requiring only a few blood samples collection is the limited sampling strategy (LSS). LSS might be developed based on multiple linear regression [98,105,113] or Bayesian estimation [71,103]. Those LSSs that include MPA concentrations within 2–3 h after drug administration might facilitate TDM. Some LSSs have been applied in clinical practice to adjust MMF dose, e.g., Berger et al. [75] conducted TDM-adjusted MMF dosing in children after renal transplantation based on LSS-calculated AUC and Carlone et al. [88] applied LSS for MPA AUC_0–12_ estimation and adjusted MMF oral dose to achieve target MPA AUC_0–12_ in children treated with MMF and Tac after HSCT.

### 3.3. Timing of Initial TDM and Target Parameters of MPA

#### 3.3.1. TDM Based on Pharmacokinetics

Based on the recommendations given for adult patients, the recommended target range of MPA AUC_0–12_ in pediatrics is 30–60 mg·h/L [65,110]; however, these values differ among MMF indications.

Filler at el. [114] suggested that MPA minimum exposure of 1.3 mg/L, which is equivalent to an AUC_0–12_ of 30 mg·h/L, may prevent the formation of donor-specific antibodies. The authors observed that those pediatric renal transplant recipients who formed donor-specific antibodies had significantly lower minimum MPA levels (0.27 ± 0.23 mg/L) than those who did not (0.47 ± 0.18 mg/L).

In children after intestinal transplantation, MPA AUC_0–12_ monitoring using a target of 30 mg·h/L contributed to the improvement of graft function and the limitation of adverse events such as renal failure [66]. In children after heart transplantation, treated with MMF and Tac, adjusted MMF dose to achieve MPA C_trough_ within 0.8–2.0 μg/mL resulted in an acceptable profile of MPA-related adverse effects without significant impact on graft outcome [82].

In children after HSCT, treated with MMF and Tac, it was proved that adjusting MMF dose to achieve MPA AUC_0–12_ within 30–60 μg·h/mL allowed a reduction in the incidence of acute and chronic GvHD [88]. Windreich et al. [87] proved that individualized MMF dosing by targeting MPA AUC was feasible, safe, and effective in the early phase after allo-HCT in children treated with MMF and CsA. In their study, optimal MPA exposure of AUC_0–24_ within 40 to 80 μg·h/mL and desired steady-state levels were achieved after switching from short infusion regimen to a continuous infusion (over 24 h). During continuous infusion, MMF dose was adjusted to achieve MPA steady-state concentration (C_ss_) within 1.7 to 3.3 μg/mL. Moreover, none of the patients showed secondary peaks what suggested the lack of enterohepatic recirculation of MPA. In children who underwent cord transplantation, Harnicar et al. [90] intensified MMF dosing from 12 h intervals to 8 h intervals to augment GVHD prophylaxis and found that patients with a mean week 1 and 2 MPA C_trough_ <0.5 μg/mL had an increased day 100 grade III and IV acute GVHD (26% vs. 9%), and those who received a low total daily MMF dose and had a low mean week 1 and 2 MPA trough had a 40% incidence of acute GVHD. The upper threshold for MPA C_trough_ was established as 2 μg/mL based on solid organ transplantation literature. The study [90] supported intensified MMF dosing and MPA trough level monitoring early after transplantation in double-unit cord blood transplantation recipients. It must be emphasized that this study included both adult and pediatric patients. In children after allo-HSCT, who received MMF concomitantly with CsA, Kim et al. [89] observed the concentrations of dose-normalized fMPA within 1 to 773 ng/mL and found that some patient-specific covariates affected unbound MPA pharmacokinetics (body weight, creatinine clearance, and total bilirubin). The desired therapeutic range of AUC_0–8_ for fMPA was assumed as 200–250 ng·h/mL.

In Sagcal-Gironella et al. study [93], MPA AUC_0–12_ ≥30 mg·h/L was associated with improved disease control; therefore, they concluded that disease activity change over time might be related to MPA exposure in childhood-onset systemic lupus erythematosus. Woillard et al. [71] suggested that a target value of 45 mg·h/L could be proposed in the context of MPA TDM in studied children. The authors observed that the risk of active disease was 21 times greater in patients with an AUC <44 mg·h/L and 59 times greater for patients with a value of AUC/dose <0.06. Similar observation was found by Godron-Dubrasquet et al. [73], who found the highest response rates of 89% in patients with MPA AUC >45 mg·h/L and concluded that this value may be considered as a target value in pediatric lupus nephritits. In their opinion, MPA TDM leading to individualized dosing may improve efficacy of MMF. Ye et al. [96] observed increased levels of MPA exposure with decreased incidence odds of diabetes, acute kidney injury, or pneumonia and proposed even higher target exposure levels of MPA AUC for clinical practice (100.39 and 50.20 mg·h/L for AUC_0–24_ and AUC_0–12_, respectively) in systemic lupus erythematosus children. In other study, Ye et al. [94] suggested that target exposure levels might amount to 98.71 and 49.36 mg·h/L for AUC_0–24_ and AUC_0–12_, respectively. Chen et al. [92] evaluated target MPA pharmacokinetic parameters using different analysis and criteria and found that in studied children, an AUC_0–12_ threshold of 39 μg·h/mL or a C_trough_ of 1.01 μg/mL was associated with the lowest risk of active disease (ROC analysis), whereas an AUC_0–12_ of <34 μg·h/mL or a C_trough_ <1.2 μg/mL (logistic regression analysis) might be associated with active disease. An AUC_0–12_ <32 μg·h/mL or a C_trough_ <1.1 μg/mL was associated with suboptimal clinical outcome, whereas an AUC_0–12_ >50 μg·h/mL or a C_trough_ >1.7 μg/mL was associated with disease control (exposure–response modeling) [92]. There has not been much information regarding the upper limit of MPA AUC, and further studies are still required in this field; however, according to Woillard et al. [71], AUC values >60 mg·h/L in children with systemic lupus erythematosus provided no additional benefit and increased the incidence of adverse drug effects.

In children with nephrotic syndrome, MPA AUC_0–12_ should exceed 45 mg·h/L [69,102] or even 60 μg·h/mL [97] to prevent from the relapse. Hibino et al. [100] postulated MPA AUC_0–24_ of 98.71 mg·h/L or AUC_0–12_ of 49.36 mg·h/L as the targeted exposure. In children with idiopathic nephrotic syndrome, Saint-Marcoux et al. [103] observed higher AUC in patients in remission (63.2 ± 27.2 mg·h/L) than in the other groups (21.3 ± 9.7 mg·h/L, 34.3 ± 18.8 mg·h/L, 30.5 ± 6.0 mg·h/L in the relapse, partial relapse, and partial remission groups, respectively) as well as higher dose-corrected AUC in the remission than in the relapse group (1.50 ± 0.61 vs. 1.1 ± 0.41). Gellerman et al. [104] found that MPA AUC_0–12_ of 60–80 μg·h/mL range seemed suitable for maintaining remission on MMF monotherapy as they did not observe a single relapse. The authors aimed at MPA C_trough_ of >2 μg/mL, which could be achieved with a dose of 1000–1200 mg/m^2^ BSA. After administrating highly variable MMF mean doses ranging from 510 to 1435 mg/m^2^/day, the mean MPA AUC amounted to 70 (range 39–113) μg·h/mL. Baudouin et al. [101] compared MPA pharmacokinetics one and six months after initiation of treatment and found its high variability and no differences between MPA C_trough_ (2.74 ± 1.16 μg/mL and 3.09 ± 1.50 μg/mL) and AUC_0–12_ (53.01 ± 21.80 μg·h/mL and 54.47 ± 16.92 μg·h/mL) in children with steroid-dependent nephrotic syndrome. MPA AUC_0–12_ values were, however, close to the upper limit of the target value for renal transplant recipients. Gellerman et al. [70] aimed at target plasma MPA C_trough_ of 1.5–2.5 μg/mL in one group and CsA target C_trough_ of 80–100 ng/mL in the second group and found that MMF might be a less nephrotoxic treatment option in children with frequently relapsing steroid-sensitive nephrotic syndrome, although MMF was inferior to CsA in preventing relapses (patients without relapse: 64% vs. 85% for MMF and CsA, respectively). In their opinion, high MPA AUC (>50 μg·h/mL) might have similar therapeutic efficacy as treatment with CsA [70]. Fujinaga et al. [115], after adjusting MMF based on MPA C_trough_, concluded that CsA appears to be more effective than MMF for maintaining remission in children with severe steroid-dependent nephrotic syndrome after a single infusion of RTX. In this study, MMF dose was adjusted to maintain MPA C_trough_ of 2–5 μg/mL, and no AUC adjustment was made, whereas CsA was adjusted to maintain C_2_ level of 400–500 ng/mL. Tong et al. [106] observed that children with MPA AUC value of only ≥30 μg·h/mL tended to require smaller steroid dosages and experience greater remission rates than patients with MPA AUC <30 μg·h/mL.

MMF was recently administered in children with Henoch-Schönlein purpura by Hackl et al. [108], who found that estimated MPA AUC_0–12_ >56.4 mg·h/L was a predictor for complete remission within 3 months. The initial dose was later adjusted according to the result of repeated TDM (performed within 3 months of therapy onset and yearly thereafter) to target MPA AUC_0–12_ values >30 mg·h/L. The authors did not encounter any adverse event requiring discontinuation of treatment during MMF treatment and concluded that MMF was a safe and potentially effective secondary treatment option for children with Henoch-Schönlein purpura to achieve and maintain long-term remission without serious side effects.

Although MPA C_trough_ generally seems not useful for TDM [71,102], some studies proved its utility in children with nephrotic syndrome. Gellerman et al. [70] found that C_trough_ levels of 3.5 μg/mL reliably predicted an AUC >50 μg·h/mL [70], whereas Sobiak et al. [97] suggested that along with MPA AUC_0–12_ >60 μg·h/mL, MPA C_trough_ >3 μg/mL may be considered as an efficient one to avoid proteinuria recurrence. In this study, MPA C_trough_ <2 μg/mL and lower fMPA were related with the risk of proteinuria recurrence. In a study by Kirpalani et al. [99], the authors calculated MPA AUC based on C_trough_ and found that in children with nephrotic syndrome, trough level monitoring may provide some information for MPA exposure if MMF administered in monotherapy. Moreover, Hibino et al. [100] suggested that MPA concentration 2 h after drug administration (C_2_) is the most useful single parameter for estimating MPA pharmacokinetics in children with clinically stable nephrotic syndrome. They generated the equation: AUC_0–12_ = 21.971 + 2.6059 C_2_ and found that target C_2_ was estimated at 10.8 μg/mL for 50 μg·h/mL of target AUC_0–12_ [100].

#### 3.3.2. TDM Based on Mycophenolic Acid Glucuronide (MPAG) and fMPA Pharmacokinetics

Hui-Yen et al. [91] suggested that mycophenolic acid glucuronide (MPAG) might be used as a simple measure of MPA metabolism and adherence; they also suggested that MPAG may be a more accurate predictor of the likelihood of response to therapy with MMF in pediatric-onset lupus nephritis. The MPAG concentrations were therapeutic (44.2 ± 26.7 μg/mL) in patients in complete remission and low (29.88 ± 22 μg/mL) in patients not in complete remission, although the difference was not statistically significant.

Although unbound, free MPA (fMPA) is the pharmacologically active form of the drug, relatively small number of studies focus on fMPA analysis [116]. Smits et al. [76] tried to answer the question whether fMPA TDM might have any advantages over current practice of monitoring total MPA. The fEC_50_ value (164.5 μL) provided an in vivo pharmacological insight into IMPDH inhibition, as its value was in accordance with the previously reported in vitro IMPDH inhibition parameters; however, it was much lower than the EC_50_ obtained from the analysis with total MPA (0.97 mg/mL). The authors stated that because only the fMPA is considered to exhibit the pharmacological effect, its parameters cannot be derived from the total MPA concentration despite good correlation between fMPA and total MPA (*r*^2^ = 0.85) [76]. Taking time and additional cost of ultrafiltration devices into account, the authors concluded that there was no clear advantage of the routine measurement of fMPA over the total MPA monitoring [76].

In a recent study of Liu et al. [116], who developed a simple and sensitive ultrafiltration and LC–MS/MS method for total and fMPA determination, the authors recommended close free drug monitoring and dose adjustments in pediatric patients with hypoproteinemia to prevent toxicity. The mean fMPA in pediatric renal transplant recipients was 0.89% (ranging from 0.62 to 1.25%), and the authors indicated that the fMPA was small and concluded that plasma albumin level plays a major role in the variability of fMPA.

#### 3.3.3. TDM Based on Pharmacodynamics

There has been still an ongoing discussion whether other biomarkers might be useful in MPA TDM. As MPA inhibits IMPDH activity, the results of such analysis may describe better the relation between MMF doses and MPA activity. There are some studies on IMPDH activity among children after renal transplantation [76,79,80,117], with nephrotic syndrome [107], and with childhood-onset systemic lupus erythematosus [93]; however, no clear recommendations have been given [79]. Dong et al. [79] observed that high pretransplant IMPDH activity was associated with high rejection rate, whereas patients with a low IMPDH baseline value experienced more adverse events. The authors did not find any age-dependent differences in IMPDH activity in pediatric renal transplant recipients; however, African-American patients appeared to have a lower baseline IMPDH activity than Caucasian patients [79]. Rother et al. [117] found large interindividual variability of IMPDH activity in both healthy children and healthy adults and no gender- and age-related differences in median IMPDH activity (82, 61, 83 and 83 μmol/s/mol AMP for healthy preschool children, school-age children, adolescents, and healthy adults, respectively). The authors found that despite a 1.9-fold increase in MPA exposure in the stable post-transplant period compared with the early post-transplant period, the corresponding IMPDH area under the activity–time curve (AEC_0–12_) decreased by only 21%. They concluded that this discrepancy might be explained by unchanged exposure to fMPA as they previously observed the increase of total MPA not fMPA in the first months post-transplant. Higher concentration IC_50_ leading to a half maximum IMPDH suppression (median, 5.4 and 4.4 mg/L for early post-transplant children and adolescents, respectively, median 9.6 mg/L for adolescents in the stable period) than previously described, found by Rother et al. [117] in children after renal transplantation, might be explained by the use of an improved assay for the determination of IMPDH activity. Higher IC_50_ corresponded with MPA C_max_ rather than MPA predose levels. Fukuda et al. [80] observed high variability of IMPDH activity and lower mean pretransplant IMPDH activity (6.4 ± 4.6 nmol/h/mg protein) in children after renal transplantation than in adults. They described the overall relationship between MPA concentration and IMPDH activity using a direct inhibitory *E*_max_ model (EC_50_ = 0.97 mg/L). In their opinion, pretransplant IMPDH activity may serve as an early marker to guide the initial level of MPA exposure required as IMPDH inhibition correlated well to MPA concentration.

#### 3.3.4. TDM Based on Pharmacogenetics

There has been a discussion on the pharmacogenetic approach to MPA TDM. A recent study conducted by Krall et al. [74] showed that CYP3A5 and UGT1A9 genotyping in pediatric recipients might be useful and advisable to guide the dosing and monitoring of MPA and Tac in children that undergo kidney transplantation. Although no significant differences between MRP2 and UGT1A9 genotypes were observed regarding most MPA pharmacokinetic parameters (C_0_, dose, dose-normalized C_0_), patients carrying the UGT1A9-275A allele had lower dose-normalized AUC_0–12_ than those carrying the UGT1A9-275T ancestral allele. In the study within pediatric renal transplant recipients, treated with MMF and Tac, Billing et al. [77] did not notice any impact of genetic variability of transporters or enzymes (CYP3A5, ABCB1, ABCG2, SLCO1B3, ABCC2, UGT1/2) on dose-adjusted MPA-AUC due to the low allele frequencies. Burckart et al. [83] proposed to include ABCC2 rs717620 polymorphisms in the pharmacogenomic analysis of outcomes after pediatric heart transplantation as they observed that the ABCC2 rs717620 AG and AA genotypes may be associated with improved, rather than poorer, rejection with hemodynamic compromise. Fukuda et al. [81] found that combined UGT1A9-440C>T, UGT2B7-900A>G, and MRP2-24T>C polymorphisms might be important predictors of interindividual variability in MPA exposure in the pediatric population.

#### 3.3.5. Other Proposed Approaches of TDM

Recently, Ye et al. [94] postulated that 25-hydroxyvitamin D levels were associated with MPA exposure levels and may serve as a potential indicator to optimize the exposure level of MPA during treatment.

### 3.4. The Optimal Dosage Regimen

For children after renal transplantation, the recommended starting dose of MMF is 1800 mg/m^2^/d when combined with CsA and 1200 mg/m^2^/d with Tac [118]. Rother et al. [117] raised the question whether dosing of MMF every 8 h might be more effective than the common twice a day regimen as they showed that IMPDH activity returned to baseline within 4–8 h of administration of MMF in children after renal transplantation. Krall et al. [74] recently published a study in which children after renal transplantation were treated with Tac and MMF at starting doses of 0.15 mg/kg twice per day and 800 mg/m^2^ twice per day, respectively. In a study conducted by Berger et al. [75], the initial dose of MMF was 400–600 mg/m^2^ orally every 12 h (up to a maximum of 2 g per day). The dose was subsequently modified to reach the target therapeutic range for MPA C_trough_ and AUC_0–12_ as 1–3.5 mg/L and 30–60 mg·h/L, respectively [75].

### 3.5. Factors Affecting Serum Concentrations in TDM

There are numerous factors influencing the pharmacokinetics of MPA, e.g., enterohepatic circulation, high degree of protein binding, drugs coadministered, time elapsed from the initiation of the therapy, and genetic polymorphism [65,67,110]. In pediatric renal transplant recipients, Smits et al. [76] observed large intrapatient and interpatient variability in binding percentages early post-transplant and at hospital discharge. The patients were treated with MMF, Tac, and corticosteroids. The binding profile became more stable over time; however, binding percentages (85.6–91.9%) were significantly lower than those reported in other patient populations. The authors listed several possibilities of their observation, e.g., excessive albumin loss through the kidney, the presence of MPAG, endogenous compounds, and comedication could compete for binding sites, decreased binding of acidic drugs (such as MPA) to albumin in chronic kidney failure, surgical stress, and reported lower albumin production [76]. In a study conducted by Kirpalani et al. [99], the authors observed a correlation between apparent MPA clearance (CL/F) and serum albumin, microalbuminuria, proteinuria, triglycerides, and hematocrit and concluded that higher drug doses may be needed to achieve adequate MPA exposure to get patients into remission [99].

Some studies proved the differences in MPA pharmacokinetics according to the age of children. It was observed that pediatric renal transplant recipients were at a greater risk for underimmunosuppression at a younger age (<6 years) due to faster clearance, even when dosing MMF per m^2^ body surface area [119]. Similarly, as in pediatric renal transplant recipients, Carlone et al. [88] found that children after allo-HSCT who were <6 years of age required a significantly higher initial MMF daily dose than older children. The difference was explained by greater drug clearance in younger children [88]. Nakaseko et al. [109] found in juvenile patients with autoimmune diseases that the actual measured MPA AUC_0–12_ and the AUC_0–12_ corrected for dose per body weight and time to reach maximum concentration (C_max_) were lower in young patients, whereas the AUC_0–12_ corrected for dose per body surface area and C_max_ were comparable among all the groups. Although Harnicar et al. [85] did not identify an age effect, they concluded that young children were potentially at special risk as patients <16 years of age had lower troughs over the 6-week period, as expected given their faster MMF metabolism. Kim et al. [84] indicated that for children <10 kg, higher doses may be required to achieve exposure similar to that in adults known to prevent acute GVHD.

Zhang et al. [85] also observed gender-related differences in pharmacokinetics. In pediatric patients after HSCT, MPA as well as MPAG plasma protein binding was significantly higher in boys compared to girls (98.3% ± 1.1% vs. 97.4% ± 1.1% for MPA; 78.7% ± 8.7% vs. 73.3% ± 9.4% for MPAG). Consequently, lower percentages of unbound forms were observed in pediatric males than females (1.7% ± 1.1% vs. 2.6% ± 1.1% for MPA; 21.3% ± 8.7% vs. 26.7% ± 9.4% for MPAG) [85].

One study described the differences in MMF dosing due to race. In children after heart transplantation, Siddiqi et al. [82] observed that higher MMF dosing was needed in African-American pediatric heart transplant recipients (702 ± 235 mg/m^2^ vs. 596 ± 99 mg/m^2^ all other races) to achieve similar MPA C_trough_.

The treatment duration is also not without significance. Windreich et al. [87] observed lower half-life and higher drug clearance in pediatric HCT recipients compared with stable pediatric renal transplant patients or adult transplant patients.

Drug–drug interactions which might have some implications for MPA TDM and have been described within the last decade concern sirolimus and nonsteroidal anti-inflammatory drugs. The interaction between MMF and sirolimus prove the importance of TDM when switching concomitant medications [68]. Alvarez-Elías et al. [68] found that patients with elevated sirolimus concentrations are at risk for MMF toxicity, whereas patients with low sirolimus concentrations would have an even greater risk for rejection because of the effect of sirolimus on the MPA exposure; however, the mechanism for this drug–drug interaction remains unclear [68]. Fukuda et al. [95] found that concomitant intake of nonsteroidal anti-inflammatory drugs may lower enterohepatic recirculation of MPA possibly through inhibition of MRP2 transport of MPAG. MMF is frequently administered concomitantly with steroids and in children after renal transplantation, the target MPA AUC_0–12_ range is 30–60 mg·h/L, it must be emphasized that for steroids free regimen, 34% higher dose-adjusted MPA AUC was observed compared to patients receiving steroids [77].

### 3.6. The Dosing in Different Groups of Patients

The initial MMF dose of 600 mg/m^2^ twice a day was administered in children after intestinal transplantation when cotreated with Tac and steroids [66]. The initial MMF dose of 600 mg/m^2^ twice a day was administered also in children after liver transplantation when coadministered with Tac or CsA [72].

MMF dosing in pediatric HCT has been primarily extrapolated from data in solid organ transplant recipients, and the optimal dosing of MMF in the HCT setting has not been clearly defined [87]. In children <12 years after double-unit cord blood transplantation, intensified dosing of 30 mg/kg/dose (nearly 900 mg/m^2^/dose) to optimize MPA exposure was proposed [90]. In children after allo-HSCT, treated concomitantly with Tac, Militano et al. [84] demonstrated that MMF administered at a dose 900 mg/m^2^/dose (maximum 1.5 g/dose) intravenously or orally in 8 h intervals was safe and associated with an improved risk of grade II–IV acute graft-versus-host disease (GVHD) as compared to 6 h intervals. In children after HSCT, other dosing was applied by Zhang et al. [85]. MMF was administered as a 2 h intravenous infusion at a dose of 15 mg/kg every 8 h concomitantly with Tac or CsA [85]. Moreover, Inagaki et al. [86] treated children steroid-refractory acute GVHD after HSCT with oral formulation of MMF at an initial median dose of 40 mg/kg/day, and the dose was increased by 1.5–2 times if manifestations of GVHD did not improve. The authors concluded that MMF was highly effective at higher doses (median maximum dose of 60 mg/kg/day) as they found no fatal toxicity as well as MMF-related infections. In pediatric-onset lupus nephritis, the daily dose of MMF was based on the renal transplant regimen of 20 to 30 mg/kg per dose or 600 mg/m^2^ per dose orally twice daily [91]. In a recent study by Chen et al. [92], in children with systemic lupus erythematosus, an initial MMF dose of 20–40 mg/kg/d twice daily (maximum of 1.5 g/d) was administered in addition to prednisolone and hydroxychloroquine. In childhood-onset lupus erythematosus, Sagcal-Gironella et al. [93] found that weight-based MMF dosing was only moderately related to MPA exposure; therefore, weight-based MMF dosing did not appear to be useful in estimating the adequacy of MPA exposure. In children with stable idiopathic nephrotic syndrome, MMF was administered twice a day at doses 30–40 mg/kg/day (maximum dose of 1000 mg/day) if coadministered with CsA [100]. Hibino et al. [100] suggested that children <6 years old require about twice the MMF dose per body weight that older children require, as the MPA exposure was lower in the youngest group. In children with frequently relapsing steroid-sensitive nephrotic syndrome and steroid-dependent nephrotic syndrome, the starting dose of MMF was 1000–1200 mg/m^2^ BSA per day in two divided doses [70,101]. Interestingly, Hackl et al. [102] observed that significantly higher daily dosage of MMF administered to patients with relapses compared with those without relapses did not result in higher MPA exposure. In juvenile patients with autoimmune diseases, the staring MMF dose was 20 mg/kg/day, and it increased to around 30–40 mg/kg/day while monitoring safety [109].

The initial dose of MMF in children with Henoch-Schönlein purpura was 800–1200 mg/m^2^ BSA/day administered in two doses [108].

### 3.7. Additional Information Useful for MPA TDM

Apart from high variability in its pharmacokinetics, the other obstacle in comparing MPA concentrations between different studies are the determination methods. The golden standard is high-performance liquid chromatography (HPLC) method, which is time- and labor-consuming and requires qualified staff. Even more accurate and precise results are obtained using liquid chromatography with tandem mass spectrometry (LC-MS/MS) method. The second method for MPA determination is the immunoassay, which is faster and easier; however, the determined MPA concentrations are 5–40% higher than those obtained using the HPLC method [110,120,121]. One must bear in mind this difference when comparing the results as in the literature both methods are used [66,67,97,100,106].

The LC-MS/MS method was also applied to determine MPA and MPAG in saliva in few studies, but only two of them included pediatric patients [122,123]. Both authors showed a significant correlation between MPA concentrations in plasma and saliva and concluded that saliva might be a suitable matrix for MPA TDM in pediatric renal transplant recipients.

Recently, Almardini et al. [78] applied dried blood spot sampling to determine MPA concentration with HPLC method. The authors concluded that using dried blood spot was a convenient direct approach to assessing adherence in children after renal transplantation and has the potential to be introduced into routine practice.

In the literature, there are some studies on generic MMF; however, most of them concerned adults. González-Ramírez et al. [124] compared the innovator product (CellCept^®^, Roche, Basel, Switzerland) and the generic (Tevacept^®^, Teva Pharmaceuticals, North Wales, PA, USA) in children with end-stage renal disease who were on the waiting list for renal transplantation. Although an important interindividual variability in bioavailability for both MPA formulations was observed, the authors found individual values of AUC within the same range as well as no statistically significant differences in bioavailability parameters between formulations. Moreover, both formulations exhibited similar drug content and dissolution profiles [124]. The authors emphasized, however, that although the generic is a suitable formulation for immunosuppressive treatment in pediatric patients, their data did not demonstrate that CellCept^®^ and Tevacept^®^ are bioequivalent formulations in children, as a lack of significant differences in bioavailability parameters is not a proof of bioequivalence.

## 4. Vancomycin (VAN)

### 4.1. Useful PK/PD Parameters

VAN is a glycopeptide antibiotic with an activity against Gram-positive bacteria. It comprises the methicillin-resistant *Staphylococcus aureus* (MRSA). It exhibits a time-dependent bactericidal effect, which means the drug’s time is above the MIC. The activity is based on disrupting the cell wall synthesis via inhibiting the incorporation of monomers into peptidoglycan chains. It is recommended for skin infections, soft tissue infections, bacteremia, infective endocarditis, pneumonia, osteomyelitis, septic arthritis, meningitis, and septic thrombosis. It is widely prescribed for hospitalized children. The pharmacodynamic parameter that describes the effectiveness of the antibacterial activity the best is the AUC over MIC [125,126].

### 4.2. Timing of Initial TDM and Target Concentration of VAN

According to the consensus recommendation, the initial doses should be calculated basing on the actual body weight. Dose adjustment should be based on serum concentration. The C_trough_ should be reached before the fourth dose at steady state conditions. The C_trough_ should be above 10 mg/L to avoid the development of *S. aureus* resistant strains. The TDM is advised for patients receiving aggressive dosing, with unstable renal function, and for patients with therapy courses longer than 3–5 days [127]. Fitzgerald et al. recommended the TDM of VAN after the first dose in the patients with cardiac arrest [128]. The MIC ≤1 mg/L is common for many MRSA strains, and the recommended C_trough_ should be within the range of 15–20 mg/L. However, the administration of VAN every 12 h with dose 15 mg/kg is unlikely to produce the concentrations of 15–20 mg/L [127,129]. For seriously ill adult patients (pneumonia, sepsis, infective endocarditis) with MRSA infections, the loading dose of 25–30 mg/kg in up to 2-h infusion with premedication of antihistamine drug is administered. The considered loading dose for seriously ill children is of 20–25 mg/kg. The targeted AUC/MIC should be >400, and C_trough_ during the VAN concentration monitoring should be within the range of 15–20 mg/L. These recommendations are valid for both pediatric and adult patients [130]. However, it is challenging because children have an increased renal clearance of VAN. It results in the C_trough_ of 10–20 mg/L, and higher doses must be administered to reach the goal concentration of >10 mg/L, which is recommended for MRSA treatment [125]. The IV dosing is 15 mg/kg/dose every six hours in children with invasive disease, bacteremia, infective endocarditis, prosthetic valve, pneumonia, osteomyelitis, septic arthritis, meningitis, brain abscess, spinal epidural abscess, subdural epynema, and septic thrombosis of cavernous or dural venous sinus. The antibiotic therapy may take even up to 6 weeks depending on the source of infection. The dose for adults is 15–20 mg/kg/dose every 8–12 h. However, it should not exceed 2 g per dose. The dose is recommended for patients with normal renal function. VAN poorly permeates the cerebrospinal fluid—1% for uninflamed and 5% for inflamed meninges. In this case, it is recommended to administer the loading dose of 15 mg/kg followed by the continuous infusion of 50–60 mg/kg/day for patients with normal renal function [130].

### 4.3. The Optimal Dosage Regimen

Frymoyer et al. [131] performed a pharmacokinetic simulation in which VAN C_trough_ of 7–10 mg/L at dose 15 mg/kg every 6 h (60 mg/kg/day) achieved the goal AUC_0–24_/MIC ≥400 at MIC 1 mg/L for MRSA. This target is easily achievable. Higher doses are not necessary. A similar conclusion was drawn by Le et al. [132], who observed that the AUC_0–24_/MIC ≥400, which corresponded with the C_trough_ within 8–9 mg/L, was achieved for the dosing regimen 60–70 mg/kg/day in 75% of patients. Higher doses than 70 mg/kg/day may be necessary for children between 1–2 years old with serum creatinine 0.2–0.4 mg/dL. The doses higher than 80 mg/kg day should be administered cautiously. It was demonstrated that weight-adjusted VAN dosing regimen should be based on age, serum creatinine, and MIC of MRSA. The dosing interval 8 h achieved similar AUC/MIC target as for the dosing interval 6 h. However, the longer time interval reduces the C_trough_ [132]. Meta-analysis performed by Moriyama et al. [133] proved that incidence of nephrotoxicity increased in the trough concentration ≥15 mg/L. The concentrations >10 mg/L allowed to attain of AUC values higher than 400 µg∙h /mL. The level of VAN ≥15 mg/L associated with kidney injury was also proved by Fiorito et. al. [134]. The necessity of the estimation of AUC_0–24_ parameter in children from intensive care units (ICU) confirmed the study conducted by Hahn et al. [135]. The patients with MRSA who required intensive ICU support had higher AUC_0–24_ and average AUC (AUC_avg_) than patients not needing the ICU support. The TDM in this group should be applied because basing only on serum creatinine measurements might not reflect VAN clearance. Clear correlation of VAN concentration with AUC does not reflect to the exposure of the drug for all pediatric patients. The best sampling to predict the values of AUC is at the end of infusion, 40–45 min after the infusion (in the distribution phase), and 12 h postinfusion or predose trough. The targeting trough levels in adult patients is simpler for clinicians. However, it is not such a simple case in children. Some children have a rapid elimination of VAN. It results in the necessity of the dose adjustment. The personalized medical treatment with the determination of AUC and MIC results in the successful therapy [136].

The dosing of vancomycin in the children with renal failure depended on the creatinine clearance. For the creatine clearance 40–60 mL/min for children <12 years, the dosing of vancomycin is 15 mg/kg IV (q8h) and for children ≥ 12 years—10 mg/kg IV (q8h). When creatinine clearance is lower—30–40 mL/min, the dosing interval changes from q8h to q12h. For creatinine clearance below 30 mL/min, the dosing interval is once a day [137].

The data concerning dosing of vancomycin in obese patients are lacking. In the meta-analysis performed by Khare et al. [138], higher vancomycin trough concentrations were found in overweight children or children with obesity. However, the heterogeneity of the published studies makes the clinical significance uncertain. The overweight and obesity may have an impact on pharmacokinetic parameters concerning distribution and elimination. The clinician should be aware that it may change the pharmacokinetic profile. In the case of adults, the dosing recommendations are clear—the dose of vancomycin must be adjusted to actual body weight [130].

### 4.4. Factors Affecting Serum Concentrations in TDM

The protein binding of VAN was approximately 50%, and it may vary between the subjects [139]. Berthoin et al. conducted a study in which the free fraction (*f*_u_) was 63.6 ± 25.8% (median 70.2%). The results comprised the range of 12–100%. The 95% confidence interval was 57.3–69.9%. A total percentage of 59% of values were outside the confidence interval [140]. De Cock et al. conducted a study of VAN protein binding in critically ill children. The unbound fraction (*f_u_*) varied between patients. The median value was 71.1% and demonstrated high variability. It ranged from 49.4% to 98.1%. The observed median C_trough_ was 6.7 mg/L [131]. Oyaert et al. conducted the study on the unbound concentration of VAN on four different patients’ populations: pediatric and adults (hematology, intensive care, and orthopedics). The unbound fraction was significantly higher for children (81.3%) than in adult patients. In hematology and intensive care populations, it amounted to ca. 61%, and for the orthopedics population, it was slightly lower (56.4%). For all investigated groups, high variability was observed, with the widest range observed for hematology patients (from 48.7 to 90.6%) [141].

The interindividual variability could also be a result of a sample preconditioning. Stove et al. conducted a study in which the unbound fraction was analyzed in patients’ samples with ultrafiltration in 4 °C and 37 °C and with equilibrium dialysis in 37 °C. The free drug fraction after ultrafiltration was 30.6% lower at 4 °C than for 37 °C. The results for equilibrium dialysis were similar to the ultrafiltration for 37 °C. VAN occurred to be stable when stored at 37 °C for 24 h. However, there were some limitations in this study, such as the pH not being adjusted, and the protocols were anonymized and some data (comedication, the VAN doses, renal function, and other patient characteristics) were not available. The model was based on the total protein concentration up to 68 g/L. It was in the lower values within the concentration range (64–83 g/L). Hypoalbuminemia is common in critically ill patients [142,143].

A study on the ultrafiltration method was also conducted by Kees et al. The authors examined the influence of centrifugal force, temperature, and pH on protein binding. The higher centrifugation force (10,000 g vs. 1000 g) resulted in shorter time to obtain the satisfactory volume of filtrate. However, the *f*_u_ of VAN was much lower for high speed of centrifugation (44.7% vs. 76.2%). The increase in pH from 6.0 to 9.0 resulted in the increase in unbound fraction from 60% to 100%. The observed *f*_u_ for pH 7.4 was 77%, and for pH 8.0, it was 82%. The increase in the pH-dependent protein binding for VAN confirmed the results obtained by Chen et al. [144]. The increase in the temperature resulted also in the increase of the *f*_u_. For the temperature range 4 °C – 37 °C, the following increase from 57% to 80% was observed. However, the difference in *f*_u_ between the room temperature (25 °C) and 37 °C was not significant (75% vs. 80%) [145].

The correct sampling procedure is important when considered the effectiveness of TDM. The venipuncture or capillary fingerstick might be considered painful and stressful. Lichlitter et al. [146] conducted a study in which the blood samples were collected by existing central venous catheter (CVC) and peripheral intravenous catheter (PIV). The measurements were conducted for VAN and tobramycin in pediatric patients. A good correlation was found between the VAN C_trough_ and random tobramycin concentration in serum for existing CVC and PIV between the concentrations obtained via peripheral venipuncture or capillary fingerstick. The limitation of the study was the fact that the correlation was found only for the above-mentioned kind of concentrations. However, the limitation of the number of venipunctures leads to the improvement of the satisfaction of patients as well as their families and medical staff.

### 4.5. The Dosing in Different Groups of Patients

McKamy et al. investigated the incidence of VAN-associated nephrotoxicity in children [147]. It occurred that the recommended C_trough_ ≥15 mg/L may be associated with a higher incidence of nephrotoxicity. The doses necessary to maintain C_trough_ on the previously mentioned level should be 50–60 mg/kg/day. The incidents of nephrotoxicity necessitated the decrease of VAN doses. Furosemide, a loop diuretic, is commonly used in the ICU. Its administration may lead to dehydration, which increase the risk of nephrotoxicity. The patients with coadministration of VAN and furosemide should be closely monitored. The aggressive dosing of VAN could also lead to drug accumulation throughout therapy. A study performed by De Cock et al. on the pediatric patients from the ICU showed that the total concentration of VAN 7 mg/L corresponded with AUC/MIC> 400 [148]. A study conducted by Giachetto et al. showed that critically ill pediatric patients on the ICU may require higher doses of VAN. It can be caused by the positive water balance and high volume of distribution. In this case, the recommended doses cause the lower concentrations, and the goal value of AUC_24_/MIC> 400 ratio will not be achieved. In this case, higher loading doses are recommended (18–24 mg/kg). The dosage should be adjusted to individual parameters [149]. Dolan et al. investigated the application of loading dose in pediatric patients. The recommended loading dose was 20–25 mg/kg. It was determined on the extrapolation of the loading dose for adults which was 25–30 mg/kg (actual body weight). The application of the loading dose was at the discretion of the clinician. The application of the loading dose improved the achievement of therapeutic concentrations; however, for the majority of patients, after receiving the loading dose, the concentrations were still subtherapeutic [130,150].

The meta-analysis conducted by da Silva Alves et al. proved that a daily dose of VAN below 60 mg/kg/day was insufficient to achieve the AUC/MIC ratio >400 or C_trough_ 10–20 mg/L. The initial dose of VAN 40 mg/kg/day may be unsatisfactory and associated with subtherapy. The dose ≥60 mg/kg/day provides effective plasma concentrations [151]. Tkachuk et al. conducted the meta-analysis on the relationship between VAN trough and AUC/MIC ratio for different pediatric hospitalized patients’ groups: general patients, cardiothoracic surgery patients, oncology patients, critically ill, and adolescents. The conducted meta-analysis for general hospitalized patients showed that the troughs between 6–10 mg/L were adequate to reach the AUC/MIC ratio above 400 for MIC 1 mg/L [126]. According to a study conducted by Benefield et al. [152] on cardiothoracic patients, the applied dosage was 20 mg/kg/dose every 8 h. Compared with the control group, the cardiothoracic surgery (CTS) patients were characterized by higher C_trough_ (18.4 vs. 8.8 mg/L). The C_trough_ within the range 10–20 mg/L were observed at >50% CTS. It was also noted that acute kidney disease developed in 25.9% of CTS patients. In the control group, it was not observed. Three other trials noted that in critically ill children, acute kidney injury (AKI) developed [153,154,155]. The incidence of AKI was from 5.4% to 17.2%. In the study conducted by Moffet et al. [153], the VAN-associated AKI occurred in 7.2% of patients. The incidence was associated with a critical illness. A retrospective cohort study conducted by Cies et al. [154] suggested no statistically significant difference in the incidence of induced nephrotoxicity with VAN in patients from pediatric ICU with VAN C_trough_ 15–20 mg/L at patients with a concentration lower than 15 mg/L. The incidence of nephrotoxicity may be associated with the duration of the VAN treatment and concomitant use of vasopressors. In a retrospective study conducted by Hays et al. [155], the VAN-associated AKI developed at 40% critically ill adolescent and young adult patients. The risk of incidence of AKI is increasing in the patients with high VAN trough levels, the concomitant use of nephrotoxic agents, and vasopressor therapy. Patients that underwent the surgical procedure were also at risk. Lanke et al. [156] conducted a retrospective study on adolescents (12–18 years old). The analysis proved that C_trough_ 10–12.5 mg/L provided the AUC_0–24_/MIC ratio ≥400. The observed MIC was ≤1 mg/L, which implies that the effective C_trough_ for adolescents are lower than for adults.

The other group in which the TDM should be introduced is children with cardiac arrest. This group is vulnerable to infections. Sepsis-associated cardiac arrest is a factor that worsens the chances of survival in patients [157]. VAN is excreted with urine, and the cardiac arrest led to the decrease of renal function and therapeutic hypothermia. Zane et al. observed that patients with normal renal function and treated with hypothermia experienced up to 25% reduction in the clearance of VAN when compared to the patients with normothermia. A reduction in clearance up to 84% was observed in patients who had reduced renal function and were treated with therapeutic hypothermia. The reduction in renal clearance results in the reduction of VAN clearance. That makes the TDM of VAN at patients with cardiac arrest treated with therapeutic hypothermia necessary [158].

Fitzgerald et al. conducted a study on children with AKI after cardiac arrest [128]. In the study, in the patients with AKI, the observed initial concentrations of VAN were higher than in those without AKI. It was a result of a lower estimated glomerular filtration rate. The C_trough_ should not be used instead of AUC to avoid the toxicity and estimate the antibiotic’s effect on children with cardiac arrest. C_trough_ in critically ill children may be found unreliable predictors of AUC, especially when renal dysfunction is observed. It makes more frequent TDM necessary, even after the first dose in all patients treated with VAN after cardiac arrest [128].

## 5. Conclusions

Recently, IFIs incidence has increased in the pediatric population, parallel with the increasing number of patients undergoing solid organ and bone marrow transplantation and those receiving chemotherapy and long-term immunosuppression. Despite sufficient care, mortality due to IFIs still remains unacceptably high. One of the possible causes of these failures is inadequate exposure to VCZ. Unfortunately, still little is known about the PK of VCZ. VCZ TDM should be routinely performed in this population to maximize the treatment effect. Large prospective studies with children are needed to further provide important information on PK VCZ and guide dosing to achieve successful treatment for pediatric patients.

Defining the target values for MPA TDM in pediatric patients is necessary for efficient treatment. Clear recommendations should be given for each of MMF treatment indication. TDM in the case of MPA is going to be still of major interest as MPA might be administered as anticancer drug due to its molecular mechanism of action: induction of apoptosis, induction of cell cycle arrest, and alteration of tumor microenvironment [159]. Moreover, there are few new immunosuppressive drugs under development for the transplant field, it is likely that MPA will continue to be prescribed on a large scale in the upcoming years [160].

The analyzed literature confirmed the necessity of TDM for VAN. The changes in elimination might be caused by the illness or the coadministration of the other drugs. The impact on water balance, elimination, and distribution may result in too high or low VAN concentration. It may have serious clinical consequences that could lead to serious adverse effects.

## Data Availability

The data presented in this study are available on request from the corresponding author. The data are not publicly available due to privacy restrictions.
